# Leptomeningeal Carcinomatosis of a Poorly Differentiated Cervical Carcinoma Caused by Human Papillomavirus Type 18

**DOI:** 10.3390/v13020307

**Published:** 2021-02-16

**Authors:** Pierpaolo Zorzato, Mattia Zambon, Silvia Gori, Helena Frayle, Maria Teresa Gervasi, Annarosa Del Mistro

**Affiliations:** 1 Department of Women’s and Children’s Health, University of Padua, 35100 Padua, Italy; pierpaolo.zorzato@gmail.com (P.Z.); mattia.zambon.4@studenti.unipd.it (M.Z.); 2 Immunologia e Diagnostica Molecolare Oncologica, Istituto Oncologico Veneto IOV-IRCCS, Via Gattamelata 64, 35128 Padova, Italy; silvia.gori@iov.veneto.it (S.G.); helena.frayle@iov.veneto.it (H.F.); 3 U.O. Ostetricia/Ginecologia, Azienda Ospedale Università Padova, 35128 Padua, Italy; mariateresa.gervasi@aopd.veneto.it

**Keywords:** cervical carcinoma, HPV18, leptomeningeal carcinomatosis, cervical cancer screening

## Abstract

Cervical cancer is caused by a persistent infection with high-risk types of Papillomaviruses (hrHPV); HPV16 and HPV18 are associated with about 70% of the cases. In the last decades the introduction of a cervical cancer screening has allowed a decrease in cervical cancer incidence and mortality; regular adhesion to the screening procedures, by pap test or HPV test, and colposcopy, according to the international guidelines, prevents cancer development and allows for diagnosis at the early stages. Nowadays, in industrialized countries, it is not common to diagnose this pathology in advanced stages, and this occurrence is frequently associated with patient’s unattendance of cervical screening programs. We describe a case of delayed diagnosis of cervical cancer, posed only after the onset of the neurological symptoms caused by leptomeningeal metastases, despite a two-year history of abnormal cytology. The endocervical mass was analyzed by immunohistochemistry, and search and typing of HPV sequences was performed by PCR in the meningeal carcinomatous cells. A poorly differentiated squamous cell carcinoma was diagnosed, and HPV18 sequences were detected. This rapidly fatal case highlights the importance of following the evidence-based recommended protocols and the preventive role of the population-based cervical cancer screening programs.

## 1. Introduction

Worldwide, cervical cancer represents the fourth most common cancer affecting women, and in Europe it is the second most common cancer in those aged 15 to 44 years [1]. It is caused by persistent infection with one of the high-risk types of Human Papilloma Virus (hrHPV), with HPV16 and HPV18 accounting for about 70% of the cases [2]. Cervical cancer can be effectively prevented [3] by population-based organized screening programs. International and national recommendations detail and update the evidence-based protocols, which actually include cytology or HPV testing performed at regular intervals, as well as management of the women with hrHPV infection and/or cytological abnormalities in order to identify and treat eventual preneoplastic high-grade cervical lesions [4]. In Italy, cervical screening was introduced in the late 1990s, and important decreases in both incidence and mortality have been obtained [5].

Invasive cervical cancer typically spreads locally via the lymphatic system to the pelvic and para-aortic lymph nodes; moreover, it can metastasize to more distant organs—commonly the lung, liver, bone, and supraclavicular lymph nodes—via the hematogenous pathway.

The five-year survival in cases of metastatic cervical cancer is only 16.5% compared to 91.5% for localized cervical cancer [6]. Early stage or locally advanced cervical cancer is treated with a combination of surgery, chemotherapy, and radiotherapy; however, there is no standard treatment for patients with metastatic cervical cancer, and the goal is usually palliative. Median survival of patients with metastatic cervical cancer is only 8 to 13 months.

Leptomeningeal metastases (LMs), characterized by the spread of cancer cells to the central nervous system (CNS) with the formation of secondary tumors within the thin membranes surrounding the brain, are a devastating complication of neoplastic diseases. The incidence of LM in cancer patients varies by the primary cancer type as well as the stage of the disease, and its occurrence is broadly estimated to range between 3 and 5%. It is considered a late manifestation of cancer and is most often diagnosed at the time of cancer relapse. Leptomeningeal metastases from solid tumors most commonly arise from breast and lung cancers and melanoma. Genitourinary cancers (including ovarian, prostate, bladder, kidney and cervical), however, rarely cause LM; in the experience of the MD Anderson Cancer Center over more than 30 years, the reported incidence of LM caused by metastatic spread of cervical carcinoma was 0.03% (4/13,289) only [7], confirmed also by literature reviews [8,9]. Poorly differentiated squamous cell and neuroendocrine carcinomas are the most common histologic subtypes of cervical cancer associated with LM [9].

We describe a case of squamous cervical carcinoma with leptomeningeal carcinomatosis diagnosed at an advanced stage to illustrate the diagnosis and clinical presentation of patients with this condition and highlight the preventive role of the population-based cervical cancer screening programs.

## 2. Material and Methods

### 2.1. Case Presentation

**Figure 1:**
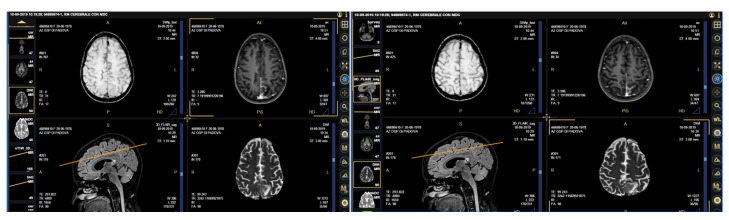
Cerebral MRI (Magnetic Resonance Imaging) highlighting the presence of leptomeningeal metastases at the bottom of the right central sulcus, on the surface of the convolutions of the left pre-wedge, with associated edema of the underlying parenchyma.

A 41-year-old woman presented to the hospital Emergency Room complaining about dysarthria with edema of the inferior arts, bilateral inguinal lymphadenopathy and headache in September 2019. The blood chemistry tests showed an evident elevation of the tumor markers CA125 (4092.6 KU/L), CA15.3 (1494.0 KU/L), and HE-4 (30.4 pmol/L). A cerebral magnetic resonance (MRI) was suggestive of a secondary lesion in the left occipital area and of meningeal carcinomatosis (Figure 1). In her anamnesis, a history of repeated visits to her private gynecologist during the previous two years was reported; the presence of atypical squamous and/or glandular cells had been repeatedly detected, with infection by HPV type 16 and type 18 in two distinct samples three and one month earlier, respectively. These findings had not been followed by an adequate management.

The patient was therefore admitted to the gynecological ward. A colposcopy revealed a bleeding portio and an invasive endocervical mass, which was biopsied and diagnosed as cervical carcinoma. A cerebrospinal fluid (CSF) sample was also obtained and disclosed the presence of carcinomatous cells. A total body computed tomography (CT) scan showed a hypodense dyshomogeneous formation in the cervix and lymphadenopathies in several districts; no other lesions were found.

The patient was transferred to the oncology ward, and palliative therapy was initiated. Her conditions worsened very rapidly; agitation and confusion began to arise, along with difficulty in swallowing, inability to walk, release of bronchial secretion with the need for repeated bronchoaspirations, epileptic tonic-clonic seizures, and stiffness of muscle tone. She died 16 days after admission.

### 2.2. Histopathology and Immunohistochemistry (IHC)

The two specimens obtained from the endocervical mass (1.2 × 0.6 × 0.3 cm^3^ and 1 × 0.3 × 0.4 cm^3^ in size) were fixed in 10% neutral-buffered formalin and embedded in paraffin; the sections were subjected to routine Hematoxylin and eosin staining. Immunohistochemical staining was also performed using primary antibodies for Melan A, S100 (targeting melanocytes), CD45 (common leucocyte antigen), AE1, AE3, MNF116 (targeting keratinocytes), and MSH2 (targeting microsatellite stability).

The cerebrospinal fluid was immediately processed; the cell pellet was sedimented on microwell slides and stained with Hematoxylin and eosin. The residual cell pellet was kept at 4 °C for subsequent analyses.

### 2.3. Search and Typing of HPV Sequences

Genomic DNA was obtained by cellular lysis of the residual carcinomatous cells contained in the CSF sample. Polymerase chain reaction (PCR) with consensus MY09/MY11 primers (targeting the L1 region; fragment length 450 bp) followed by restriction fragment length polymorphism (RFLP) analysis was performed, as previously described [10]. These primers are able to detect a wide range of HPVs, encompassing high- and low-risk types, known to have a good capacity to detect the types predominantly associated with cervical cancer [11]. DNA amplificability was evaluated by PCR with GH20/PC04 primers for the beta-globin gene (fragment length 268 bp). PCRs with type-specific primers for HPV18 (targeting the E7 region, fragment length 104 bp) and for HPV16 (targeting the E6 region, fragment length 323 bp) were also performed. Positive and negative controls were included in each PCR run and in the RFLP analyses; DNA from the HPV16-positive SiHa and the HPV18-positive HeLa cervical cancer cell lines, and no DNA, were used, respectively.

## 3. Results

### 3.1. Pathological and Immunohistochemical Findings

**Figure 2:**
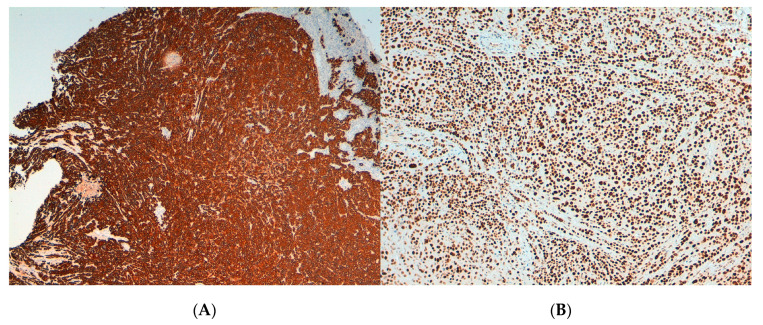
Microphotographs showing the immunohistochemical staining of the endocervical mass compatible with poorly differentiated squamous carcinoma. (**A**) MNF116: positive (**B**) MSH2: positive.

The biopsies obtained from the endocervical mass showed cervical tissue infiltrated by poorly differentiated squamous cells. The immunohistochemical analyses were negative for Melan A, S100 and CD45, and positive for AE1, AE3 MNF116 and MSH2 (Figure 2). A diagnosis of poorly differentiated squamous cell carcinoma was established.

**Figure 3:**
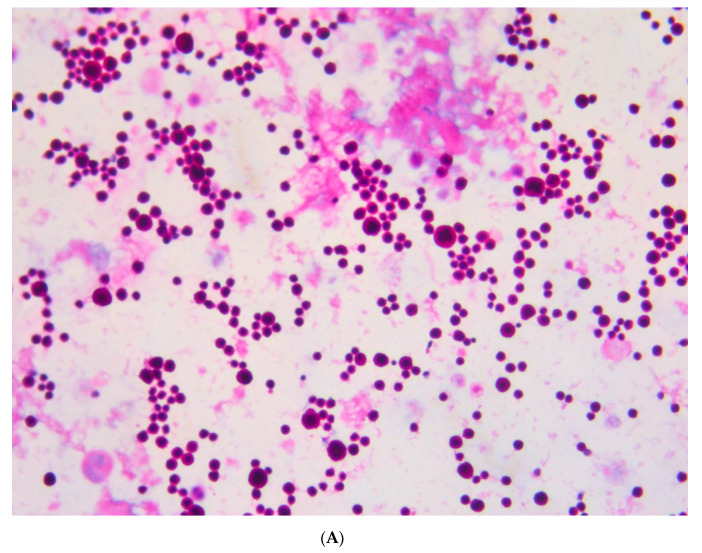

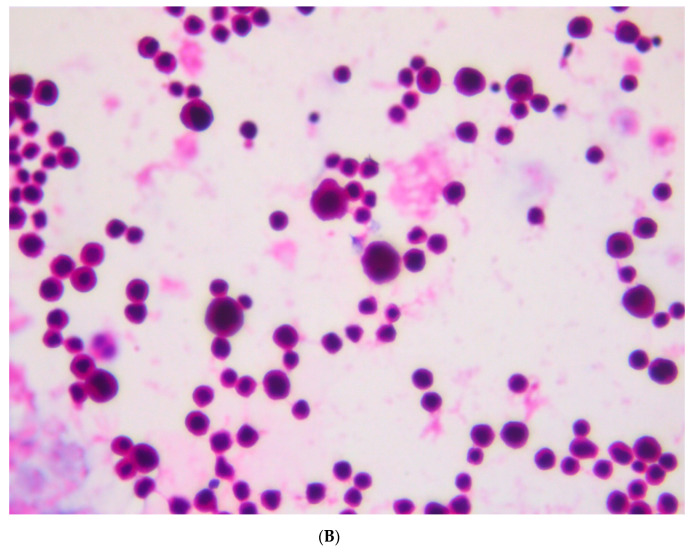
Cerebrospinal fluid cytology examination: massive presence of carcinomatous cells. Hematoxylin and Eosin staining; magnification 20× (**A**) and 40× (**B**).

The examination of the cerebrospinal fluid (CSF) sample disclosed a massive presence of carcinomatous cells characterized by highly heterogeneous size and hyperchromatic nuclei (Figure 3).

### 3.2. Virological Findings

The DNA extracted from the carcinomatous cells contained in the CFS was strongly positive for both the beta-globin gene and HPV sequences. RFLP analysis of the MY09/MY11 amplified fragment revealed positivity for HPV sequences of type 18. The presence of type 18 was further confirmed by amplification with the HPV18 type-specific primers, while no amplification was obtained with HPV16 type-specific primers. This result confirmed the finding of HPV18 infection detected shortly before admission to the hospital.

## 4. Discussion

The squamous cell carcinoma of the cervix is a cancer effectively preventable by population-based organized cervical cancer screening programs, which operate by active invitation of women in the target age range (25 to 64 year-old in Italy) to periodic visits and include evidence-based quality-controlled protocols for the management of the women with positive screening test, in order to rule out or diagnose and treat preneoplastic lesions. In fact, cytology every three years is performed in 25 to 29 year-old women, and hrHPV testing, with cytology triage in cases with positive hrHPV test, every five years in 30 to 64 year-old women. Subsequent referral of the women with hrHPV infection and/or cytological abnormalities depends on the lesion’s risk related to the specific clinico-pathological and virological findings; women with hrHPV infection and abnormal cytology (ASC-US+) undergo immediate colposcopy, with biopsy of suspicious areas; women with hrHPV infection and normal cytology undergo repeat hrHPV testing one year later, with colposcopy in case of persistent infection. If no high-grade lesions are detected at colposcopy, women are invited to additional visits according to follow-up protocols [12]; persistent hrHPV infection and high-grade cytology are associated with the highest risk of a high-grade lesion’s development and mandate a strict monitoring.

In our patient’s anamnesis there was a history of abnormal pap smears in the last two years; atypical glandular cells (AGC) and atypical squamous cells—cannot exclude high-grade (ASC-H) [13]—were repeatedly diagnosed; the last pap smear had been performed two months before admission to the hospital. Moreover, positivity for HPV18 had been documented in cervical cells collected one month earlier. These visits had always been performed by a private gynecologist, who omitted to perform the appropriate exams to investigate those findings, while the patient had never attended the organized cervical cancer screening program. Indeed, the high positive predictive value (PPV) for high-grade lesions of ASC-H (and to a lesser extent of AGC) mandates the need to perform colposcopy, taking biopsy of any suspicious area, and to investigate the endocervical canal [12,14]. In the patient of our case report, the positivity for HPV18 added additional risk for the presence of a (pre)neoplastic lesion. This HPV type is detected in a significant proportion of invasive carcinomas (and particularly of adenocarcinomas), but is much less frequent in the preneoplastic high-grade lesions [15,16,17,18,19]. Although it is not yet clear whether this might be linked to a different natural history of cell transformation or to a high rate of progression to invasion of such lesions, it implies a peculiar difficulty in the prevention of HPV18-associated lesions, particularly by cytological screening [20]. HPV18 belongs to the alpha-7 species group, together with types 39, 45, 59, and 68; interestingly, other two cases of central nervous system (CNS) involvement by cervical cancers related to HPV18 and HPV45, respectively, have been reported [21,22], suggesting the possibility of a higher predilection for cerebral and leptomeningeal disease by alpha-7 HPV types.

Meningeal metastasis has been postulated to occur after spread to the lungs. This is supported by reports that the lungs are the most common area for metastatic cervical cancer; in addition, this pattern of spread is typical in other types of systemic cancers, such as lung cancer, breast cancer, and melanoma. However, there were some reported cases of patients with intracranial metastases from cervical cancer without lung metastases [9].

In our case, the diagnosis of cervical cancer was posed only after the onset of the neurological symptoms, with carcinomatous meningitis becoming the initial presentation, a rare occurrence of a very rare manifestation [23]; the devastating disease progression hampered any therapeutic intervention and caused her death in about two weeks, a time in line with the reported median survival from LM diagnosis of few weeks to few months [24].

In conclusion, our case report highlights the occurrence of a very rare clinical presentation of an invasive cervical squamous cell carcinoma that could have been prevented or diagnosed at an earlier stage by the correct application of existing evidence-based protocols. The important role of the organized cervical cancer screening, performed in a setting assuring quality monitoring, for effective prevention of this disease is also underlined. Indeed, the World Health Organization (WHO) promoted in 2020 an action to eliminate cervical cancer, by the worldwide application of anti-HPV vaccination, cervical screening, and treatment of precancerous lesions and invasive cancer [25,26].

## Data Availability

Not applicable.
